# Decoding oxygen prescriptions: electronic health record documentation versus patient-reported use

**DOI:** 10.1186/s12890-024-03248-7

**Published:** 2024-10-08

**Authors:** Wilson Tang, J. Smith, J. Dakkak, A. Balasubramanian, B. Seth, C. Leotta, S. C. Mathai, M. C. McCormack, S. Acharya, A. Calypso, S. K. Danoff

**Affiliations:** 1 The Johns Hopkins University, Baltimore, MD USA; 2 The Johns Hopkins University School of Medicine, Baltimore, MD USA

**Keywords:** Long-term oxygen therapy (LTOT), Oxygen prescription, Electronic Health Record (EHR), Oxygen management communication

## Abstract

**Background:**

Long term oxygen therapy (LTOT) is prescribed for hypoxemia in pulmonary disease. Like other medical therapies, LTOT requires a prescription documenting the dosage (flow rate) and directions (at rest, with activity) which goes to a supplier. Communication with patients regarding oxygen prescription (flow rate, frequency, directions), monitoring (pulse oximetry) and dosage adjustment (oxygen titration) differs in comparison with medication prescriptions. We examined the communication of oxygen management plans in the electronic health record (EHR), and their consistency with patient-reported LTOT use.

**Study design and methods:**

A cross-sectional study was conducted in 71 adults with chronic lung disease on LTOT. Physician communication regarding oxygen management was obtained from the EHR. Participants were interviewed on their LTOT management plan. The information from each source was compared.

**Results:**

The study population was, on average, 64 years, two-thirds women, and most used oxygen for over 3 years. Only 45% of both at-rest and with-activity oxygen prescriptions were documented in the Electronic Health Record (EHR). Less than 20% of prescriptions were relayed to the patient in the after-visit summary. Of those with EHR-documented oxygen prescriptions, 44% of patients adhered to prescribed oxygen flow rates. Nearly all patients used a pulse oximeter (96%).

**Interpretation:**

We identified significant gaps in communication of oxygen management plans from provider to patient. Even when the oxygen prescription was clearly documented, there were differences in patient-reported oxygen management. Critical gaps in oxygen therapy result from the lack of consistent documentation of oxygen prescriptions in the EHR and patient-facing documents. Addressing these issues systematically may improve home oxygen management.

**Supplementary Information:**

The online version contains supplementary material available at 10.1186/s12890-024-03248-7.

## Background

Over 1.5 million Americans are prescribed long-term oxygen therapy (LTOT) for chronic heart and lung conditions [1]. Initiation and maintenance of oxygen prescriptions rely on measurements of oxygen saturation (SpO_2_) typically during ambulation on a flat surface during clinical visits. These ambulatory saturations may vary with disease progression. They may also fail to capture real world oxygen requirements with activities such as walking up steps or carrying loads. Thus, additional modifications may be required to meet a patient’s dynamic daily needs [2].

The delivery and management of LTOT is complex and often influenced by a network of stakeholders including clinical providers, patients, payers, oxygen equipment manufacturers, durable medical equipment providers (DMEs), and pulmonary rehabilitation providers [1]. In a survey of 1,926 patients with chronic pulmonary conditions and oxygen-dependence, patients reported a lack of oxygen management plans or education, with only 8% of respondents reporting receiving healthcare provider instruction on home oxygen therapy [3]. While oxygen education may be available through clinics and advocacy groups, patient confusion and misunderstanding regarding self-management of oxygen and equipment usage persist [3]. This lack of clarity in oxygen prescription and management, ultimately contributes to decreased satisfaction with oxygen, poor adherence, and decreased quality of life [4].

Prior studies revealed clinician instruction improves oxygen adherence. However, physician oxygen prescriptions are often limited. In a review of > 500 patients, only 16% had adequate oxygen prescriptions [5, 6]. Further, there is limited data on patient understanding of and adherence to oxygen therapy and whether this aligns with provider prescriptions for LTOT. Hence, we sought to (1) evaluate provider documentation of oxygen management plans in the Electronic Health Record (EHR) system, (2) evaluate communication of oxygen management plans to patients in EHR and (3) assess patient-reported understanding of the oxygen prescription, adherence to that prescription, and other patient usage and practices.

## Methods

### Study design and population

Participants were recruited from three subspecialty pulmonary clinics- Interstitial Lung Disease (ILD), Chronic Obstructive Pulmonary Disease (COPD), and Pulmonary Hypertension (PH)- at a single tertiary care hospital from 2/6/2019 to 10/13/2019. Inclusion criteria were use of long-term oxygen treatment (LTOT) for greater than one month in the outpatient setting, while receiving care by a pulmonologist within the Johns Hopkins Hospital system, age over 18 years old, English-speaking, and the ability to provide written informed consent. The interview was completed orally, often with participant’s partner or caregiver present, and a study team member recorded participant responses in real time. A complete ‘oxygen management plan’ was defined as documentation of all aspects of oxygen care: oxygen flow rates, SpO_2_ targets, titration goals, and devices (e.g., tank, oxygen concentrator, liquid oxygen and the oxygen delivery mode (continuous vs. pulse)). This ‘oxygen management plan’ was assessed in both physician documentation and communication to the patient, as well as patient understanding using the patient’s electronic health record (EHR) and interview responses respectively. Congruence for various categories was assessed by comparing information across EHR and interview data on a per-patient basis.

### Electronic Health Record (EHR) analysis

Three types of documentation were reviewed to investigate patients’ oxygen management plan from the EHR (EPIC Hyperspace August 2021 Release, Epic Systems Corp., Johns Hopkins Hospital): (1) Home Oxygen Order (HOO) (official EHR documentation of oxygen prescriptions), (2) Physician Encounter/Visit Notes with mentions of “oxygen” or “O_2_”, and (3) After-Visit Summaries (AVS) (EPIC-generated summaries of visit and prescription information generated after each visit). These three documents provided a comprehensive picture of (1) the official O_2_ prescription, (2) visit documentation and information discussed, and (3) written information relayed to the patient respectively. The following data were abstracted for analysis: oxygen flow rates and modality, SpO_2_ targets, titration strategy, and device data. The majority of flow rate modality across EHR documents was not specified (Table 1) explicitly as continuous (‘C’) but were written in a continuous format (L/min) and considered continuous for analysis. In contrast, pulse (P) flow for portable oxygen concentrators was denoted as an arbitrary setting. For titration strategy, the notes were reviewed for any mention of titration strategies conveyed to the patient. SpO_2_ targets alone were not considered sufficient for a titration strategy since they lacked additional language and guidance on whether to titrate to reach this SpO_2_ target. In the 2 cases of minor differences between the three documents, the official home oxygen order data was used for analysis.

**Table 1:** Interview Study Population characteristics

Characteristics	All Respondents*	Interstitial Lung Disease (ILD)	Chronic Obstructive Pulmonary Disease (COPD)	Pulmonary Hypertension (PH)
Total	71	42 (59%)	25 (35%)	20 (28%)
Sex:				
Female Male	47 ( 66%) 24 (33%)	26 (62%) 16 (38%)	17 (68%) 8 (32%)	13 (65%) 7 (35%)
Age (mean ± sd**)**:	64 +/- 11	61 +/- 11	67 +/- 10	64 +/- 11
Portable Device Use*:				
Gas Cylinder Liquid Vessel Portable Oxygen	42 (59%) 8 (11%) 44 (62%)	27 (64%) 4 (10%) 27 (64%)	14 (56%) 3 (12%) 14 (56%)	10 (50%) 2 (12%) 12 (60%)
Concentrator (POC)				
Years on LTOT:				
< 1 year 1–3 years 3 + years	13 (18%) 21 (30%) 37 (52%)	10 (24%) 13 (31%) 19 (45%)	4 (16%) 6 (24%) 15 (60%)	3 (15%) 5 (25%) 12 (60%)
Insurance*:				
Private Medicare Medicaid None	49 (69%) 47 (68%) 6 (1%) 1 (< 1%)	30 (71%) 29 (69%) 3 (7%) 1 (2%)	15 (60%) 16 (64%) 3 (12%) 0 (0%)	15 (75%) 12 (60%) 0 (0%) 1 (5%)
Flow Rate L/min (Median, IQR) *N* = 70:				
At-Rest With-Activity	2.75 (2.0, 4.0) 3.0 (3.0, 5.0)	2.5 (2.0, 4.0) 4.0 (3.0, 5.0)	2.5 (2.0, 4.0) 4.0 (2.0, 5.0)	3.0 (2.0, 4.0) 4.0 (2.0, 6.1)

* Participants reported more than a single entry for devices, diseases, and insurance

### Interview development

The interview content was developed iteratively through discussion with pulmonologists with expertise in ILD, PH and COPD (SD, SCM, AB, MC) as well as a nurse practitioner (AC) and respiratory therapist and piloted by 13 patient participants. The interview included 51, multiple choice and short-answer questions. (Supplement 1- Survey Text) Data included duration of oxygen therapy, as well as flow requirements at rest and with activity. Information regarding daily oxygen adjustments, implementation of LTOT management plan, and usage of pulse oximetry were also obtained.

### Statistical analysis

Statistical analyses were conducted using Python 3.6.8 (Python Software Foundation, https://www.python.org) and Stata 16 (StataCorp, College Station, TX USA). Summary statistics for participant characteristics were described using means with standard deviations or medians with interquartile ranges for continuous variables. Categorical variables were described with proportions, and percentages. Data were compared on a per patient basis across their EHR data and interview responses. Differences in reported flows greater than 0.5 L/min and flow modality (pulse versus continuous) were considered in disagreement, or incongruent, as were any differences in target SpO_2_.

## Results

### Interview responses and general characteristics

The study population consisted of 71 respondents with COPD, PH, or ILD using LTOT under the care of 22 pulmonologists in the Johns Hopkins Hospital System. Prevalence of pulmonary diagnoses were 59% ILD, 35% COPD, and 28% PH, with a minority of individuals having more than one of these diagnoses (Table 1). The average age of respondents was 64 years old and 66% were women (Table 1). The majority (52%) reported using oxygen for more than 3 years. In addition to portable oxygen equipment, 90% of participants used a stationary oxygen device at home. Using our original definition, 33 (46%) participants reported all aspects of a defined oxygen management plan in their interview responses. All 71 patients responded to the titration strategy question but only 33 patients had an actual strategy; the remainder did not titrate their oxygen.

When reporting flow rates, participants initially reported a with-exertion flow rate as continuous flow rate in L/min. However, when asked specifically about their portable device, they reported a 0.5 median (IQR 0.5–1.5) pulsatile flow setting lower than their response in LPM.

### Electronic Health Record (EHR) documentation

Three types of documentation were reviewed: the official EHR “Home Oxygen Order”, the “Physician Encounter/Visit Notes”, and “After Visit Summaries’’. Each provided different parts of the oxygen management plan (see Table 2). Overall, “Home Oxygen Orders” were the most consistent, frequently covering 3 of the 4 categories in the oxygen management plan (Flow Rates, SpO_2_, and Device). Of the 822 after visit summary documents (EPIC-generated After Visit Summary), authored by 54 unique providers (including non-pulmonary providers), only 19% mentioned oxygen therapy at all. Less than 15% contained prescribed oxygen flow rates in the format of L/min or LPM. Twenty-six participants had at least one AVS with specific flow rate documentation and 6 patients had a target SpO_2_, but the data were different across documentation. None of the data sources captured titration strategy, and all generally lacked details of the O_2_ management plan compared to the interview response data. Using our original definition, there were no patients with all aspects of the defined oxygen management plan documented primarily due to a lack of documented titration strategies. Only 28 patients (39%) had complete documentation of flow rate, SpO_2_, and device.

**Table 2:** Summary of Oxygen Management Plan Data

	Electronic Health Record (EHR)					Interview		
Type of Data	Home Oxygen Order	Visit/ Encounter Notes	After Visit Summaries	EHR Total		Interview Response	Percentage Incongruent (N)	
Flow Rate: *At Rest* Total (C/P)	26 (25/1)	31 (29/2)	26*	36	42	71 (71/0)	50% (15)	56% (18)
*With-Exertion* Total (C/P)	33 (31/2)	35 (31/4)		38		71 (32/39)	66% (25)	
SpO2 Goal	28	0	6	28		71	86% (24)	
Titration Strategy	0	0	0	0		33	N/A	
Device	22	27	0	30		71	53% (14)	

* Inclusion of the entire oxygen prescription was assessed in after visit summaries. C-continuous, P-pulse

### Oxygen Management Plan: interview response and comparison with EHR

#### Oxygen flow

From the interview responses, all patients reported an oxygen prescription for both at-rest and with-activity flow rate, with a median of 2.75 L per minute^1^ (LPM) at-rest (IQR: 2.0–4.0), and 4.0 LPM with-activity continuous flow rate (IQR: 3.0–5.0).^1^ However, a majority (55%) of patients reported using a pulse delivery portable oxygen concentrator and at a median pulsatile flow setting of 3.5 (IQR 2.75–4.0), which is lower than the continuous flow reported.

In the EHR, a specific prescribed flow rate was documented for 42 of the 71(59%) participants. Of these 42 patients, 32 had both at-rest and with-activity flow documentation, 4 patients had only at-rest flow rates, and 6 patients had only with-activity flows. The median at-rest flow rate found was 2.0 LPM (IQR: 2.0–4.0) while the median with-activity flow rate was 3.0 LPM (IQR: 2.0–4.0).

When compared on a case-by-case basis across continuous flow responses, the majority (24/42) 57% of patients reported flow rates that were incongruent with their prescription. For at-rest continuous flows, 50% of patients reported incongruent flow at a mean of 0.48 +/- 1.6 LPM more than prescribed oxygen. For with-activity continuous flows, 66% (25/38) of patients reported incongruent flow, using on average 0.78 +/- 1.3 LPM more oxygen. While there were only 4 pulse prescriptions documented, all corresponding patients reported a continuous flow prescription. The majority, 55% (39/71), of patients also reported using a pulse device and flow settings that were lower by a median of 0.5 than their continuous flow.

#### Patient Oxygen Titration Strategy

**Fig. 1:**
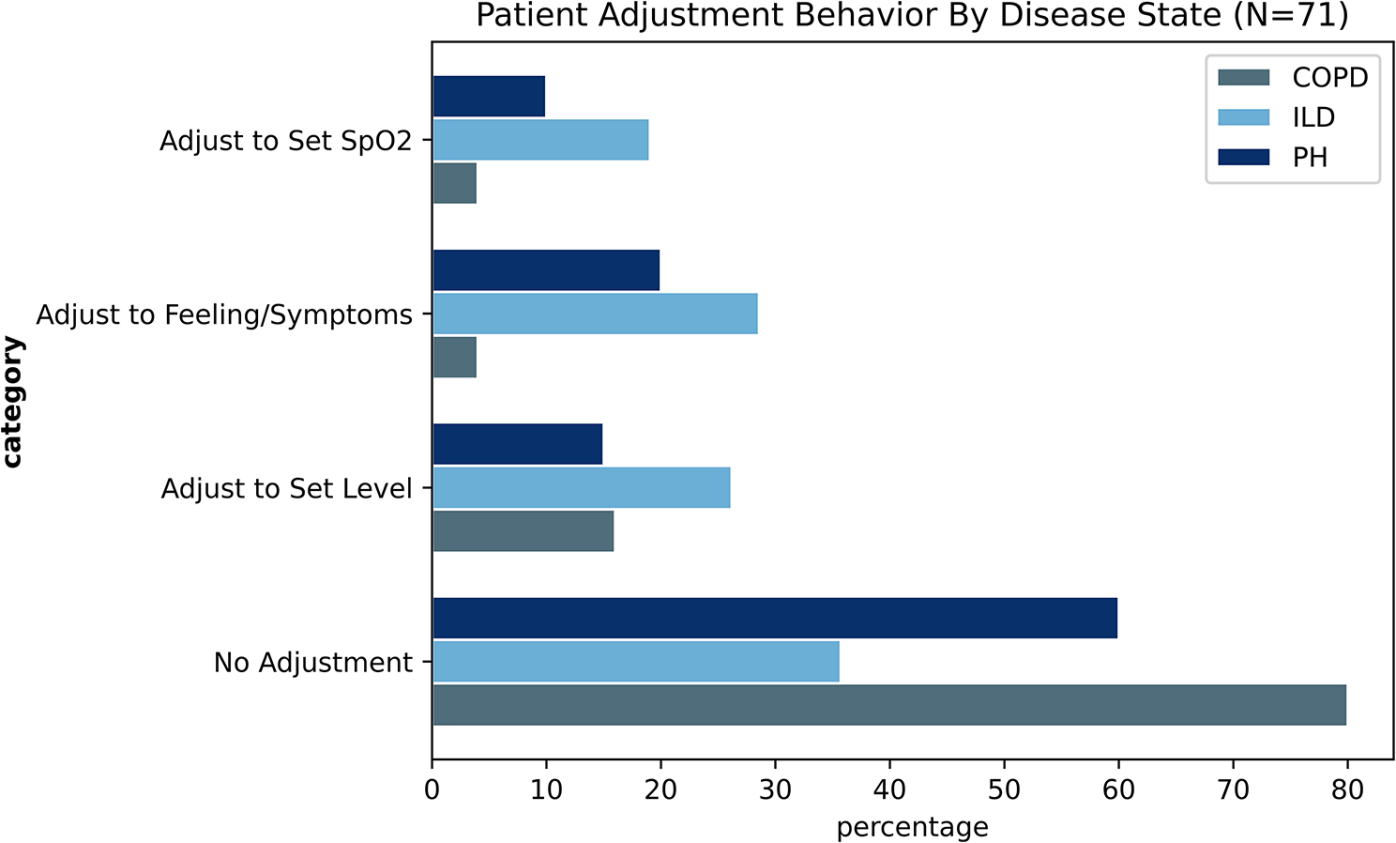
Patient Adjustment Behavior Responses by Disease State

While the EHR had no mention of an explicit titration strategy, 46% [*n* = 33/71] of participants’ interview responses indicated that they had some titration strategy. Common strategies for adjusting oxygen flow included to “Feel Better” or “Eliminate Immediate Symptoms” [*n* = 14/70] (20%), followed by “Adjusting to a Predetermined Oxygen Level” [*n* = 13/70] (19%). Across disease states, COPD was the least likely to use any strategy (Fig. 1). When asked about pre-emptive oxygen adjustment strategies, 51% of respondents [*n* = 36/71] stated that they turned up the flow rate on their equipment in anticipation of an increase in need for oxygen or to prevent symptoms. When analyzed across disease states, individuals with ILD (60%) were more likely to adjust their oxygen flow rate throughout the day [*n* = 25/42] compared to 20% of COPD [*n* = 5/25] or 30% of PH [*n* = 6/20].

**Fig. 2:**
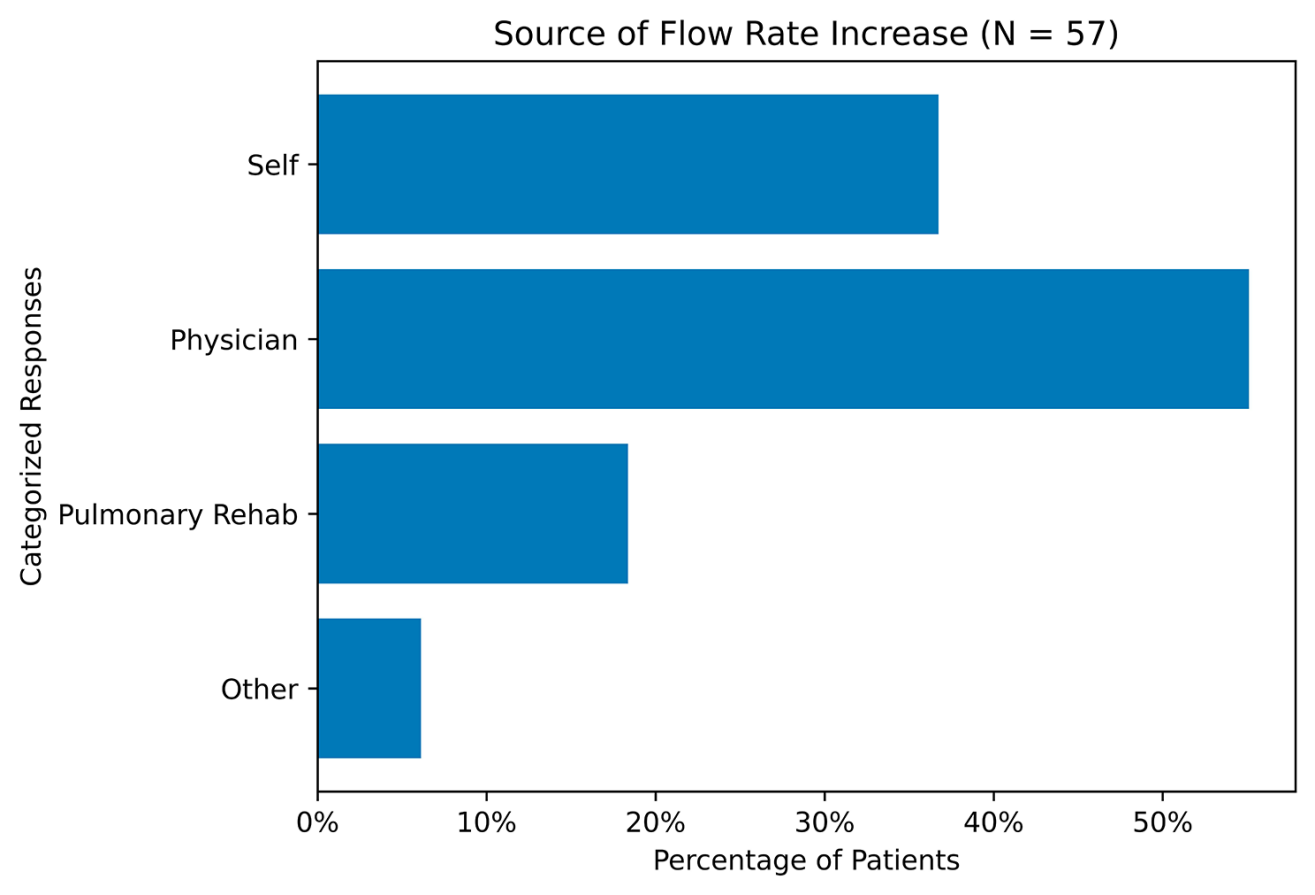
Patient Reported Source of Flow Rate Increase

Fifty seven of the 71 participants responded to a question about the source of the most recent increase in baseline oxygen flow rate. The most common response was “Physician” [*n* = 27/57] (55%), then “Self-titration” [*n* = 18/57] (37%), followed by “Pulmonary Rehabilitation” [*n* = 9/57] (18%) (Fig. 2).

#### SpO2 monitoring and targets

**Fig. 3:**
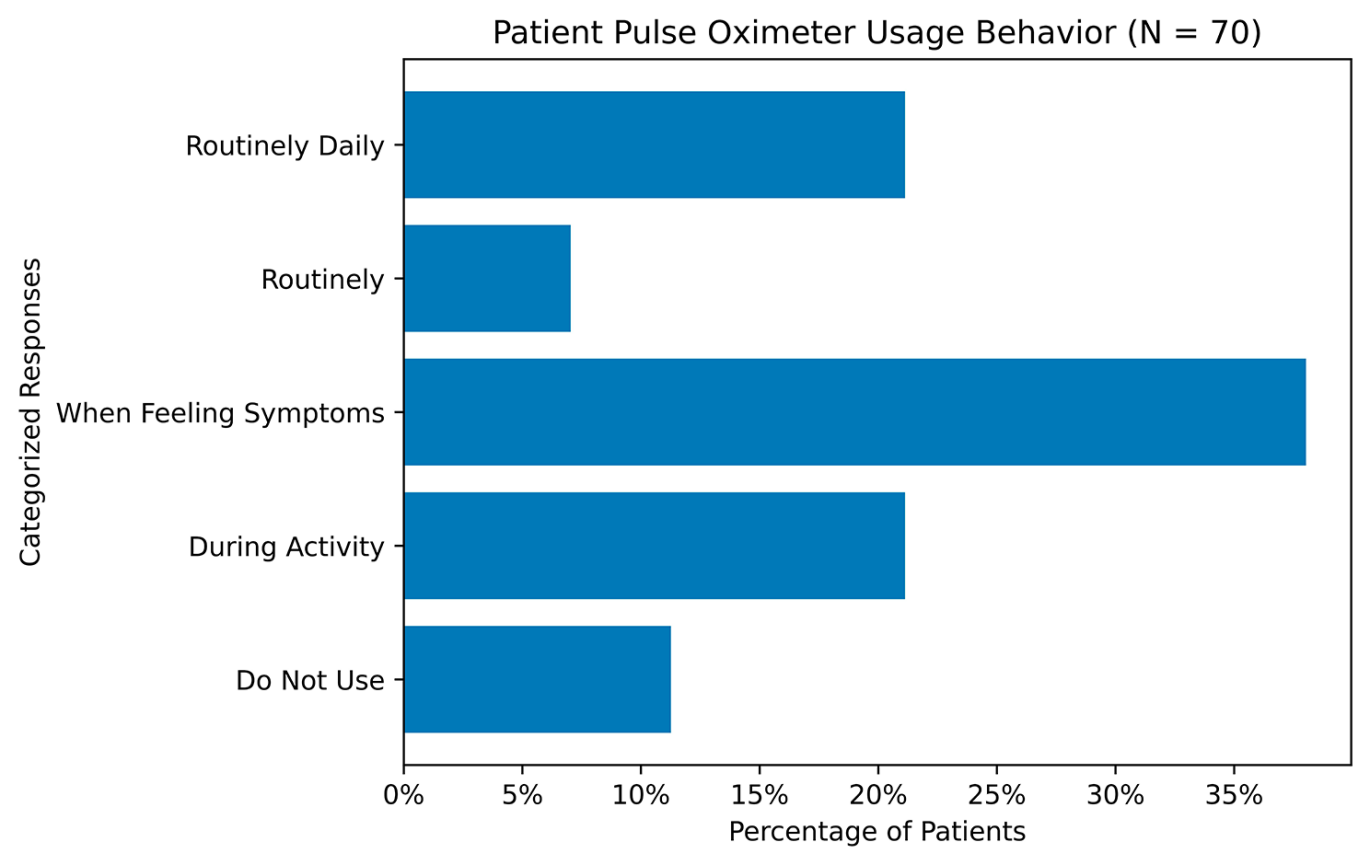
Patient Reported Pulse Oximeter Usage Responses

The majority of participants (89%) [62/70] indicated using a pulse oximeter in their LTOT management. The frequency and indication for use of the pulse oximeter varied across respondents, including regular usage at timed intervals (daily (15/70, 21%; as part of a routine, 5/70, 7%), as well as conditional use such as during activity (15/70, 21%) and when feeling symptoms (27/70, 38%) (Fig. 3). Among pulse oximeter users, 82% [58/62] indicated having a target SpO_2_ during activities. Participants with at least one situational SpO_2_ targets [*n* = 62] aimed to stay above 92% (+/- 4) SpO_2_ during normal activity[*n* = 58], and targeted [*n* = 61] 91% (+/- 4) during exercise.

Corresponding data on SpO_2_ goals from the EHR was limited: only 28 participants had EHR records with a goal SpO_2_ from 88 to 92%. These SpO_2_ goals were not contextualized by activity levels unlike those reported during the interview. Patient-reported SpO_2_ goals across activity types were 2.7% points higher than their EHR records.

#### Device

All patients reported a portable oxygen device during their interview with the most common equipment being a “Portable Concentrator” at 62% (*N* = 44/71), “Portable Gas Oxygen Tank” at 59% (*N* = 42/71), and “Portable Liquid Oxygen Tank” at 11% (*N* = 8/71). Only 30% (*N* = 21) of patients reported more than one portable device.

Of the 71 participants, a specific portable device for oxygen therapy was documented in their EHR for 29 patients (41%). The most common equipment listed was a “Portable Gas Oxygen Tank” at 52% (*N* = 15/29), followed by “Portable Concentrator” at 45% (*N* = 13/29), and followed by “Portable Liquid Oxygen Tank” at 7% (*N* = 2/29).

When comparing their interview and EHR documentation, 16 of the 30 patients (53%) patients reported their EHR device in their interview response. Of the 14 patients with incongruence, the most common difference (50%) was the usage of a “Portable Concentrator” while their EHR stated a “Portable Gas Tank” or “Portable Liquid Tank” in 7 patients, followed by the 6 patients (43%) who had the inverse case, and 1 patient using a “Portable Liquid Oxygen Tank” despite being prescribed a “Portable Gas Oxygen Tank”.

## Discussion

This study aimed to assess EHR-based physician documentation and communication of outpatient oxygen therapy prescriptions and compare to patient self-reported O_2_ management. By studying patients on O_2_ with several advanced lung disorders (ILD, COPD, PH) we were able to identify common threads in the experience of O_2_ usage independent of diagnosis or specific provider. Further, by focusing on a single tertiary care population, we were able to interrogate a common EHR system (EPIC) and evaluate granular, patient-level information in the EHR as well as obtaining corresponding patient experience through the survey. We found significant gaps in oxygen documentation and communication from healthcare providers in a commonly used EHR system (EPIC Hyperspace August 2021 Release, Epic Systems Corp., Johns Hopkins Hospital). There was poor documentation of the actual O_2_ prescriptions including flow rate, device and context (at-rest and/or with-activity), and flow modality.

**Fig. 4:**
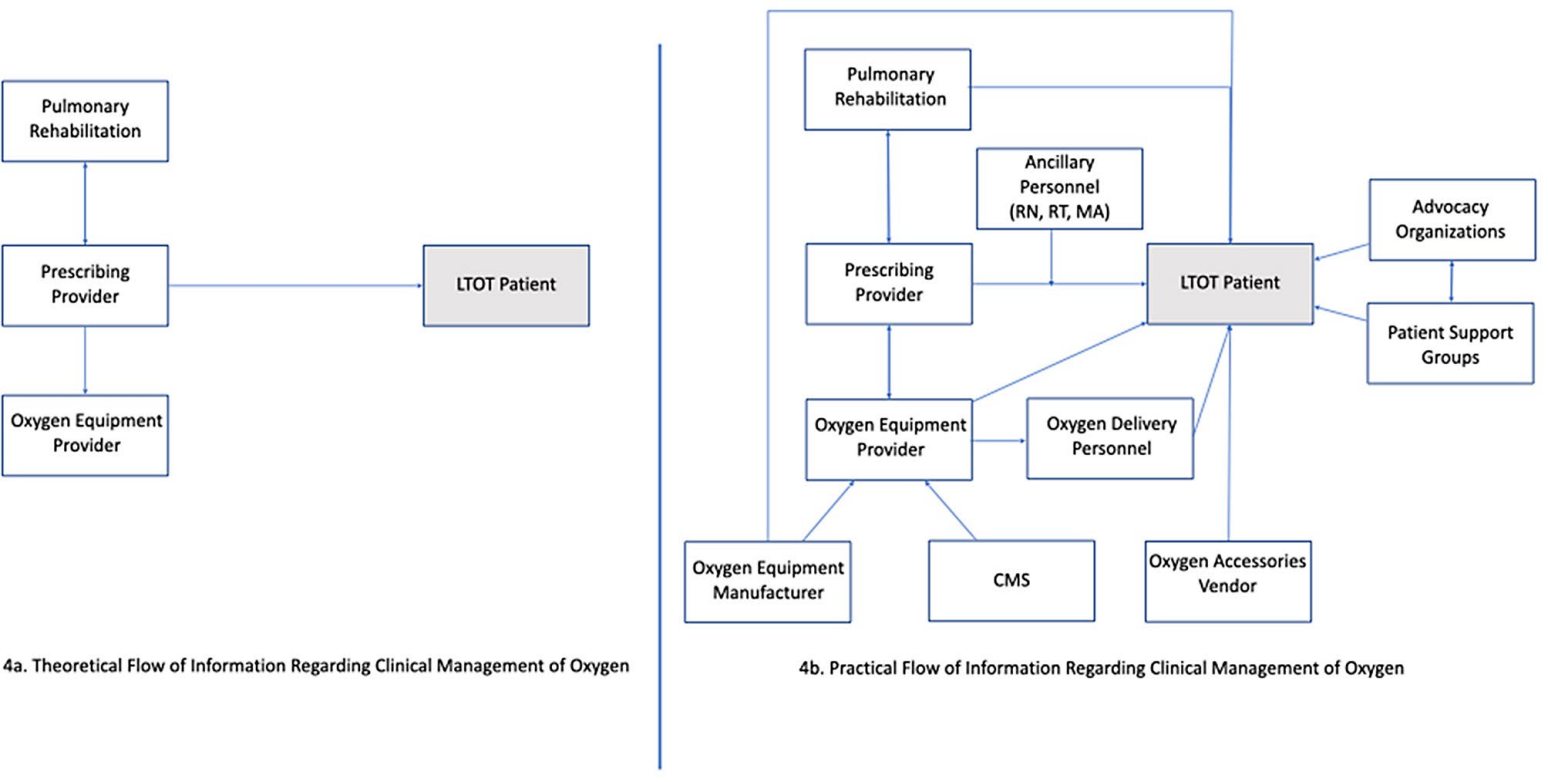
Theoretical versus Practical LTOT Patient Information Map and Flow

The complicated and fragmented oxygen ecosystem (Fig. 4), often results in lack of physician understanding of oxygen equipment or assumptions that others in the system will be handling documentation, leading to gaps in documentation. Further, O_2_ titration strategies were limited and poorly communicated to the patient (as evidenced by the lack of this information in the AVS provided to patients). In the absence of this documentation and communication, patients improvised with an O_2_ titration strategy. There are a number of possible reasons for the lack of titration strategy documentation. The Center for Medicare and Medicaid (CMS) does not require a titration plan to be included in the prescription. Further, it may be difficult for a provider to provide a titration strategy for patients based on a limited ambulatory desaturation test on a flat surface. We consistently found incongruence between EHR documentation and interview responses for at-rest (50%) and with-activity (66%) oxygen flow rates, flow modality (55%), SpO_2_ saturation goals (86%), and even devices (53%). These findings highlight key gaps in current systems for documentation and communication of oxygen management plans, and thereby identify opportunities to improve EHR documentation and patient communication of a complete and optimal LTOT management plan.

During our review of patient EHR documentation, we found relevant oxygen records in 3 categories: flow rates, SpO_2_ goal and device. However, the EHR did not automatically relay this information to the patients as it does for medication prescriptions, with fewer than 20% of EHR-generated After Visit Summaries containing oxygen prescription information. This lack of hard-wired communication suggests that oxygen prescriptions are considered different or perhaps less significant compared to medications. This may provide a plausible explanation for the incongruence seen between EHR and interview data. The EHR system (EPIC) used did not categorize oxygen orders and associated information in a manner similar to prescription medications. Prescription medications, their doses and instructions for consumption are automatically included in the After Visit Summary. However, oxygen related information must be manually added as a note. This is a missed opportunity to provide critical oxygen information to the patient. A similar lack of physician-patient information flow was documented in a national survey of oxygen patients, where > 60% respondents reported receiving home oxygen education from the delivery personnel vs. only 4–9% from a healthcare professional [3, 7].

In an ideal situation, like medication prescriptions, complete information would be provided by the healthcare provider directly to the patient verbally and in writing as well as to others in the care pathway (DME, pulmonary rehabilitation). However, in the current system, LTOT users can receive information regarding oxygen management from upwards of 4 stakeholders in addition to their prescribing clinician, each with limited ownership (Fig. 4). It is important to note that CMS does not dictate oxygen device’s portability requirements to the DMEs, leading to a gap between patient’s requested and the DME provided device. The fragmented communication leaves the patient to determine optimal home oxygen dosing with limited support [8, 9].

These communication challenges translate to variable self-management strategies across patients and disease states. Only 44% of participants used their prescribed flow rates during daily activities. While O_2_ titration was utilized by 46% in the overall study population, 60% of the ILD population reported at least some titration, compared to 30% and 20% in PH and COPD respectively. The reason for this trend is unclear. It may be related to the more acute nature, and high-flow rates that ILD patients often need. Among those who do adjust their oxygen flows, the most common strategies included adjusting until symptoms subsided (20%) or adjusting to achieve a predetermined oxygen saturation level (18%).

For those who did use pulse oximetry regularly, most participants reported checking SpO_2_ only when symptomatic, which may miss periods of asymptomatic hypoxemia [10]. This emphasizes the need for education on use of pulse oximetry, especially in patients with resting hypoxemia While almost all patients used a pulse oximeter, only 23% of patients reported using a pulse oximeter in their typical daily self-management. A prescribed SpO2 goal was documented in only 45% of oxygen orders. Only 11% of patients reported not using a pulse oximeter at all, which corroborates national rates. In a survey of 1926 participants, 93% of patients reported using pulse oximeters [3]. However, the low rate of prescribed SpO2 targets in the EHR and in After Visit Summaries means that patients have limited physician-sourced information to guide them.

The lack of consistent documentation and communication (via AVS) of Oxygen Management Plans as well as the lack of congruence between EHR documentation and actual patient day-to-day practices, should be alarming given the importance of oxygen therapy in this chronic lung disease population. Without proper documentation and communication surrounding this crucial therapy it is no surprise that the patient’s experience with oxygen therapy is often a negative one. Addressing these issues may improve the management of oxygen and patient perception of oxygen therapy.

## Conclusion

Inconsistent documentation and communication of O_2_ prescriptions to the patient in the EHR is problematic. While there are many gaps in this system, the failure of the EHR system to manage oxygen in a manner similar to other prescription medications and the resulting lack of patient facing documentation may worsen communication gaps for patients on LTOT. The evidence of communication breakdown at various levels presents opportunities for a multi-level approach to enhance oxygen-related communication and management. This study also points to the need for further system level evaluation of oxygen prescription practices and use among individuals on LTOT.

Our work identifies a need to improve the physician-patient communication and EHR documentation process for LTOT. This may be achieved by either including oxygen prescriptions in the medication list, which enjoys a higher level of support for AVS and patient communication, or directly improving the current systems for oxygen prescription in the EHR via further integration of HOO and DME orders in the AVS and other patient communication systems. These orders should translate to patient communication in the after-visit summary and include details pertinent to device settings (continuous oxygen flow rates or pulse dose number setting) during rest, exertion, and sleep titration strategies, and oxygen saturation targets at-rest, and with-exertion.

Further investigation of other EHRs and health systems is needed to validate the documentation and communication gaps observed in this cohort, and to define opportunities to improve oxygen delivery at the health system level.

### Limitations

There are several limitations to this study. First, there is a potential lack of generalizability as respondents were a convenience sample of LTOT users from a single tertiary care center. This was necessary to allow for both the granular evaluation of EHR documentation and corresponding participant interviews. While the study population may not be fully representative of LTOT users with advanced lung disease, the complexity of the oxygen prescription pathway described in this cohort is also reflected in larger national surveys. Secondly, the ability to participate in the interview may reflect greater interest in clinical resources related to oxygen use, better healthcare access and/or socioeconomic status. Further, participants may over-report their own behaviors as a desirability bias. Third, as only English-speakers were included in the study population, there may be an even greater barrier in patient understanding and implementation of their oxygen prescription in an non-English speaking population. Fourth, as with prior research [4], the majority of respondents were women, who may not be representative of the sex distribution of patients with chronic lung diseases on LTOT. We also had a higher representation of ILD patients in our cohort, rather than COPD patients who nationally represent the largest population of patients on LTOT, however, systems for oxygen prescription and device provision remain the same regardless of disease. Fifth, documentation and correspondence regarding oxygen prescription and management are not standardized throughout the medical record, potentially limiting the accurate assessment and interpretation of the exposure of oxygen order communications. Finally, iterations of the EPIC EHR are not necessarily consistent across health systems, which could impact the generalizability of these results.

## Electronic supplementary material

Below is the link to the electronic supplementary material.

Supplementary Material 1

## Data Availability

There are two datasets generated and/or analyzed during the current study. The survey dataset can be made available from the corresponding author on reasonable request. However, the Electronic Health Record (EHR) Data is not publicly available due to specific individual patient EHR data, which is proprietary to Johns Hopkins.

## Footnotes

1 3 patients indicated 2.5, 2.5, and 6.5 LPM as their flow rate for at-rest.

## Declarations

### Royalties or Licenses

Up to Date (Authorship and editorial work for pulmonary function testing). MM.

### Consulting Fees

Boehringer Ingelheim (Advisory Board PF-ILD, SSc-ILD), Lupin Pharma (Advisory Board ILD) SD.

Aridis, GlaxoSmithKline, NDD Medical Technologies, MCG Diagnostics, Boehringer Ingelheim (Consulting) MM.

### Payments for Expert Testimony/Meetings

Boehringer-Ingelheim (Lecture Travel) SD.

Talem Health (Medical Education) MM.

### Data Safety Monitoring or Advisory Board:

Galecto DSMB Galactic Trial, Galapagos DSMB ISABELA Trial. SD.

Janssen, United Therapeutics, Merck, Acceleron SCM.

Pulmonary Fibrosis Foundation (Senior Medical Advisor), American Thoracic Society (Board). SD.

### Leadership or Fiduciary Role in other board:

Patient Centered Outcomes Research Institute Rare Disease Advisory Panel. SCM.

## Abbreviations

LTOT: Long Term Oxygen Therapy
COPD: Chronic Obstructive Pulmonary Disease
ILD: Interstitial Lung Disease
PH: Pulmonary Hypertension
EHR: Electronic Health Record
AVS: After Visit Summary
HOO: Home Oxygen Order
DME: Durable Medical Equipment
SpO_2_: Peripheral Capillary Oxygen Saturation
CMS: Center for Medicare & Medicaid Services
