# Supplementary material for: Decoding oxygen prescriptions: electronic health record documentation versus patient-reported use
Source: BMC Pulm Med. 2024 Oct 8;24:491. doi: 10.1186/s12890-024-03248-7 (PMC11460145; doi:10.1186/s12890-024-03248-7)
Supplement: Supplementary file 1 — Supplementary Material 1 [file 12890_2024_3248_MOESM1_ESM.docx]

**CBID Pulmonology 2019 Patient Survey: LTOT (IRB00196054) Consent Form**

This survey is being conducted through the Department of Biomedical Engineering at The Johns Hopkins University. We are a group of graduate students at The Johns Hopkins Center for Bioengineering Innovation and Design working to improve the standard of care within pulmonology. Your experiences and input will aid in designing a new method of portable oxygen supplementation in order to better meet the clinical needs and lifestyles of patients.

Your completion of the survey will serve as your consent to be in this research study. The survey is expected to take approximately 30 minutes. All responses will remain anonymous and will only be shared for research purposes.

______________________________________

Patient Name (printed)

______________________________________ _____________________________

Patient Signature Date

**CBID Pulmonology 2019 Patient Survey: LTOT (IRB00196054)**

This survey is being conducted through the Department of Biomedical Engineering at The Johns Hopkins University. We are a group of graduate students at The Johns Hopkins Center for Bioengineering Innovation and Design working to improve the standard of care within pulmonology. Your experiences and input will aid in designing a new method of portable oxygen supplementation in order to better meet the clinical needs and lifestyles of patients.

Your completion of the survey will serve as your consent to be in this research study. The survey is expected to take approximately 30 minutes. All responses will remain anonymous and will only be shared for research purposes.

**General Questions**

*The following questions relate to your general lung condition, therapeutic oxygen usage, mobility, and quality of life.*

1. What is your date of birth?___________________
2. What is your sex?___________________
3. What state do you reside in?___________________
4. Are you currently covered by medical insurance? Circle all that apply.
   1. Medicare
   2. Medicaid
   3. Private Insurance
   4. None
5. If covered by private insurance, who is your insurance provider?___________________
6. What is your respiratory diagnosis? Circle all that apply.
   1. COPD
   2. Interstitial Lung Disease
   3. Pulmonary Hypertension
   4. Other___________________
7. How long have you been using supplemental oxygen?

___________________years ___________________ months

1. How many hours a day do you use oxygen therapy
   1. 0-5 hours/day
   2. 6-11 hours/day
   3. 12-17 hours/day
   4. 18-24 hours/day
   5. Other

1. What was your initial prescribed flow rate (L/min) when you were first prescribed oxygen?
   1. Minimal Flow Rate: ___________________L/min
   2. Maximal Flow Rate: ___________________L/min
2. What is your current prescribed flow rate (L/min)? * (Both at-rest and with-activity)
   1. Minimal Flow Rate: ___________________L/min
   2. Maximal Flow Rate: ___________________L/min

*If answers to Q8-9 are different:*

1. How did you learn you had to increase your flow rate? Circle all that apply.
   1. Self-determined
   2. Physician recommendation
   3. Pulmonary rehabilitation
   4. Other ___________________
   5. N/A

Comments: __________________________________________________________________

1. What symptoms do you experience during daily activities (i.e. showering, washing dishes, etc.)? Circle all that apply and rate the severity on a scale of 1-5, 5 being the most severe.
   1. Shortness of breath: N/A___1___2___3___4___5
   2. Mucus production: N/A___1___2___3___4___5
   3. Cough: N/A___1___2___3___4___5
   4. Increased heart rate: N/A___1___2___3___4___5
   5. Dizziness: N/A___1___2___3___4___5
   6. Other___________________: N/A___1___2___3___4___5
   7. Other___________________: N/A___1___2___3___4___5
   8. Other___________________: N/A___1___2___3___4___5
2. When experiencing symptoms, how do you adjust your oxygen equipment to the appropriate flow settings? *(e.g. always use pulse oximeter, increase flow until I feel better, etc)*
3. What sources of oxygen supplementation do you use? Circle all that apply. ** (No pulse conservers for tanks were observed)

See chart with oxygen equipment

- 1. Stationary Concentrator
  2. Portable Gas Oxygen Tank
  3. Portable Liquid Oxygen Tank
  4. Portable Concentrator
  5. Other ___________________

1. With your portable devices, do you preemptively increase your oxygen to prevent possible symptoms or in anticipation of increased activity?
   1. Yes
   2. No

Comments: __________________________________________________________________

1. With your portable devices, do you immediately turn your oxygen flow rate back down when your symptoms subside or when you decrease your level of activity?
   1. Yes
   2. No

Comments: __________________________________________________________________

1. At your current flow rate, do you humidify the oxygen from your device?
   1. Yes; If yes, how? ____________________
   2. No
2. Do you require any of the mobility aids listed below? Circle all that apply.
   1. Cane
   2. Crutches
   3. Walker
   4. Manual wheelchair
   5. Electric wheelchair
   6. Motorized scooter
   7. Other ___________________
   8. N/A
3. Is your mobility assistance due to limitations in oxygen technology? If so, how?
4. How do you rate the overall burden of using and transporting each type of oxygen equipment you own?
   1. Stationary Concentrator

_____No burden

_____Minimally Inconvenient

_____Inconvenient

_____Strenuous

_____Extremely Strenuous

- 1. Portable Gas Oxygen Tank

_____No burden

_____Minimally Inconvenient

_____Inconvenient

_____Strenuous

_____Extremely Strenuous

- 1. Portable Liquid Oxygen Tank

_____No burden

_____Minimally Inconvenient

_____Inconvenient

_____Strenuous

_____Extremely Strenuous

- 1. Portable Concentrator

_____No burden

_____Minimally Inconvenient

_____Inconvenient

_____Strenuous

_____Extremely Strenuous

- 1. Other ___________________

_____No burden

_____Minimally Inconvenient

_____Inconvenient

_____Strenuous

_____Extremely Strenuous

Comments:

1. Who supplies your oxygen equipment (Durable Medical Equipment Provider)?
2. Do you require assistance transporting your oxygen equipment?
   1. Yes
   2. No

Comments: __________________________________________________________________

1. What are the greatest challenges and limitations you face with your oxygen equipment?
2. If we were to solve one issue with your oxygen supplementation, which one would make the biggest impact to you and how?

**Portable Concentrator**

*Improvements in technology have now enabled ambient air to be concentrated to pure oxygen for clinical use. This technology is called a portable concentrator. This device is electrically powered and requires the use of batteries when on the go.*

*The following questions relate to your portable oxygen concentrator usage and battery life of your device, if applicable.*

1. Do you have a portable concentrator?
   1. Yes
   2. No

*If answer to Q24 is No:*

1. If you do not have a portable concentrator, why? Select all that apply:
   1. Cost
   2. Availability
   3. Technology does not meet my oxygen needs
   4. I do not feel the need for a portable concentrator

Other ___________________

**** If you do not have a portable concentrator, skip to Design Improvements section. ****

1. How did you obtain your portable concentrator? Circle all that apply
   1. Purchased out of pocket
   2. Entirely covered by insurance
   3. Partially covered by insurance
   4. Rental
   5. Other ___________________
2. What is the brand and model of your portable concentrator?
3. What type of flow setting do you use?
   1. Continuous Flow
   2. Pulse
   3. Both

*If answer to Q28 is Continuous Flow or Both:*

1. What flow rate(s) (L/min) of oxygen do you set your portable concentrator to when using continuous flow?
   1. Minimal Flow Rate: ___________________L/min
   2. Maximal Flow Rate: ___________________L/min

*If answer to Q29 is Pulse or Both:*

1. What level(s) of oxygen do you set your portable concentrator to when using pulse flow?
   1. Minimal Flow Rate: ___________________L/min
   2. Maximal Flow Rate: ___________________L/min
2. How has a portable concentrator affected your mobility and independence?
3. Have you ever used a portable concentrator though you knew you required more oxygen than it could provide? If so, why?
4. On average, how long does your concentrator battery typically last without recharging or being replaced?

____________h____________mins

1. How many additional batteries have you purchased since the initial purchase of your portable concentrator?
2. How many extra batteries do you typically take with you when leaving the home?
3. What additional supplies have you purchased for your portable concentrator, if any?
4. Overall, how much have you spent on additional supplies for your portable concentrator?

**Design Improvements**

*Although portable concentrators are linked to greater mobility and independence, limitations still remain withflow rates (1-3 L/min), weight (4-20 lbs), and battery life (1-6 hrs). New research is focused on surpassing these limitations and providing more efficient oxygen supplementation.*

*The following questions relate to the use of pulse oximetry, nasal cannulas and overall goals of future oxygen supplementation equipment.*

1. What type of pulse oximeter do you use? Circle all that apply.
   1. Finger
   2. Ear
   3. Nose
   4. Other ___________________
   5. N/A
2. When do you use a pulse oximeter, if ever?
3. What is your goal blood oxygenation level (%SpO_2_) for the following scenarios?
   1. Sleeping___________________
   2. Mostly sitting___________________
   3. Mostly walking___________________
   4. Performing any higher level of activity___________________
4. *Some patients find it challenging to maintain healthy oxygenation levels, since they can vary so drastically with activity. This can lead to unexpected symptoms.*

Would a device that automatically adjusts the flow of oxygen for you based on your oxygenation levels (%SpO_2_) be beneficial to you? Why or why not?

1. If your oxygen could be automatically adjusted for you through a pulse oximeter, what device(s) would you be willing to continuously wear:
   1. Finger pulse oximeter
   2. Ear pulse oximeter
   3. Finger or ear pulse oximeter
   4. I would not be willing to wear a pulse oximeter continuously
2. Have you ever used a high-flow nasal cannula? See image of high flow cannula
   1. Yes; what was your experience with it?
   2. No

Comments:

*Current oxygen technology requires a trade-off between the weight of the equipment and the amount of time oxygen can be provided. Although lighter equipment is easier to carry, it provides oxygen for a shorter amount of time.*

1. How much does your typical portable oxygen setup weigh?
2. How do you transport your portable oxygen setup?
   1. Carry
   2. Wheel
   3. Other___________________
   4. N/A

1. If your portable setup could last an additional hour, how much additional weight would you be willing to:

Carry?_________________________ Wheel? _____________________

1. What is the maximum weight of a portable device are you willing to: Carry?_________________________ Wheel? _____________________
2. What is the minimum amount of time you require from your oxygen technology outside of the home?
3. Would you be willing to tolerate more noise from your cannula if it meant your portable oxygen could last longer?
   1. Yes
   2. No
4. Current concentrators can cost between $2,000-$4,000. If there was a portable concentrator that could provide the flow rates you need, what is the maximum you would be willing to pay out of pocket (no insurance coverage)?
   1. I would not pay out of pocket
   2. Under $1,000
   3. $1,000 - 2,000
   4. $2,000 - 3,000
   5. $3,000 - 4,000
   6. $4,000 - 5,000
   7. Above $5,000

*Thank you very much for your response! The responses provided for this survey will aid in designing a new method of portable oxygen supplementation in order to better meet the clinical needs and lifestyles of patients. In order to create an impactful product, we need your input. If you would like to participate in future research and development of the product, please check the box below and provide your email. Your information will remain confidential and used for only this research purpose.*

☐ I would like to be contacted regarding future research for the development of a more portable oxygen concentrator.

Email:______________________________________
